# On the Fluid Dynamical Effects of Synchronization in Side-by-Side Swimmers

**DOI:** 10.3390/biomimetics4040077

**Published:** 2019-12-05

**Authors:** Ramiro Godoy-Diana, Jérôme Vacher, Veronica Raspa, Benjamin Thiria

**Affiliations:** Laboratoire de Physique et Mécanique des Milieux Hétérogènes (PMMH, UMR 7636), CNRS, ESPCI Paris–PSL University, Sorbonne Université, Université de Paris, F-75005 Paris, France; jerome.vacher@espci.org (J.V.); veronica.raspa@gmail.com (V.R.); bthiria@pmmh.espci.fr (B.T.)

**Keywords:** collective swimming, bio-inspired swimmers, fluid–structure interaction, self-propulsion, synchronization, fish schooling

## Abstract

In-phase and anti-phase synchronization of neighboring swimmers is examined experimentally using two self-propelled independent flexible foils swimming side-by-side in a water tank. The foils are actuated by pitching oscillations at one extremity—the head of the swimmers—and the flow engendered by their undulations is analyzed using two-dimensional particle image velocimetry in their frontal symmetry plane. Following recent observations on the behavior of real fish, we focus on the comparison between in-phase and anti-phase actuation by fixing all other geometric and kinematic parameters. We show that swimming with a neighbor is beneficial for both synchronizations tested, as compared to swimming alone, with an advantage for the anti-phase synchronization. We show that the advantage of anti-phase synchronization in terms of swimming performance for the two-foil “school” results from the emergence of a periodic coherent jet between the two swimmers.

## 1. Introduction

The interaction between neighboring individuals constitutes the fundamental fabric of collective dynamics [1]. Depending on the system, this interaction can be established by many different kinds of sensing and actuation, involving for instance optical, chemical, magnetic, or mechanical signals [2,3,4], and the collective response can emerge from nonlinear feedbacks based on local interactions [5]. In the case of swimmers (see, e.g., [6] for a review on sensing and actuation in fish inspired robots), biological or artificial, regardless of the kind of sensing, their interaction is mediated by hydrodynamics. The understanding of interactions between neighbors is not only crucial for the understanding of fish schooling, but also in the case of robotic swimmers, where one would like to have accurate models or design guidelines for the optimal behavior of a robot in the presence of neighbors. Concerning fish schools, it is challenging to include hydrodynamic interactions in models with large numbers of swimmers without resorting to simplified theoretical approximations, e.g., [7,8]. On the other hand, zooming into the problem of a single swimmer, despite the successes of Lighthill’s theory to describe the mechanics of fish swimming [9], the ubiquitous problems of the interaction between a swimmer and its environment are out of its reach. This environment can be a wall, a substrate (a significant number of works have explored this problem in different contexts [10,11,12,13]), or another neighboring swimmer. The interactions between multiple swimmers may significantly impact the performance or cost of locomotion associated with fish schooling, as each swimmer moves in a non-uniform and unsteady flow created by its neighbors. Collective effects in fish swimming have been widely studied theoretically/numerically, e.g., [14,15,16], and experimentally [17,18,19]. The goal of the present work is to examine the hydrodynamics of a basic experiment of the interaction between two neighboring swimmers, which we model as undulating flexible foils.

**Figure 1:**
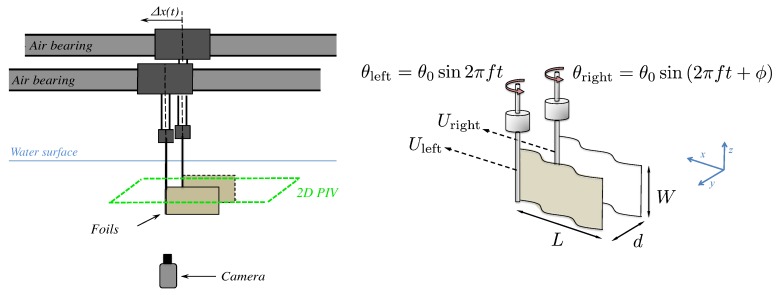
Schematic diagrams of the experiment with two self-propelled swimmers in a side-by-side configuration. Each foil is held by an independent air-bearing rail (**left**) and actuated by an oscillating pitching motion of an axis that holds the foil from one extremity (**right**).

Actuated flexible foils have been extensively studied in the last decade as a model of fish-like swimmers or as a bio-inspired propeller, and their dynamical behavior is ruled by a complex fully-coupled fluid–structure interaction problem, e.g., [20,21,22]. In this paper, we report on an experiment with two self-propelled independent swimmers, each one constituted by a flexible foil actuated by pitching oscillations at one extremity, placed in a side-by-side configuration with imposed swimming direction (see Figure 1). We have already used different versions of the experiment described here, on the one hand to examine the dynamics of a single swimmer [22,23], and on the other hand to consider the effect of swimming near a wall [12]. In the latter, we established the main physical mechanism that leads to a performance enhancement due to the presence of the wall: a reorientation of momentum.

Conceptually, the setup with two swimmers that we study in the present paper is one of the simplest model realizations of the minimal school [24,25] and has been designed to examine the complex flow dynamics that arises due to the undulation kinematics of the two swimmers. Recent experiments on real fish in a swimming channel with imposed velocity have shown that fish favor a synchronized kinematics with their nearest neighbors as the swimming speed of the school increases [17,18]. That observation has brought evidence to previous numerical predictions on the hydrodynamic advantages of synchronized swimming [16] and is the seed for the specific objective of the present paper: to compare the flow dynamics and the performance of the pair of interacting swimming foils in the two possible states of synchronization: in-phase and anti-phase kinematics. In order to focus on this comparison, we picked a single point in the kinematic, geometric, and material parameter space for the case of an elastic foil actuated by pitching oscillations, thus fixing several parameters that define the problem.

## 2. Experimental Setup

Following [23], the experiments were carried out in a free-surface water tank (0.9 m × 0.8 m × 0.5 m) with no external flow where two swimmers were fully submerged and allowed to move freely in a rectilinear motion, independently, using an air-bearing rail to hold each swimmer (see Figure 1). Each swimmer was made of a thin rectangular foil (length L=0.15 m, span W=0.1 m, and thickness 130 μm, made of Mylar) of bending rigidity $B=1.02\times 10^{-3}$ N m. The foil was clamped to a cylindrical axis (d=0.005 m in diameter) that constituted the head of the swimmer and set the swimming depth in the water bulk. A pitching motion was imposed, which generated self-propulsion by creating a backward propagating undulation along the flexible body. The rotational oscillation of the head of each swimmer was controlled with 0.5 degrees of precision by a small stepper (see Figure 1) and a sine wave driving curve so that:

$$\theta_{\mathrm{left}}=\theta_{0}\sin2\pi ft\;\;\;\mathrm{and}\;\;\;\theta_{\mathrm{right}}=\theta_{0}\sin2\pi ft+\phi \;.$$

**Figure 2:**
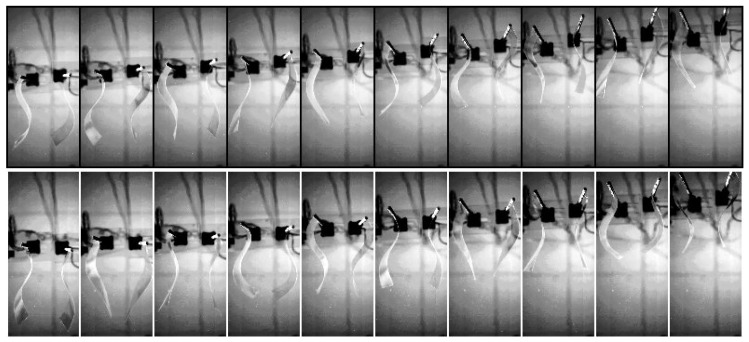
Two image sequences are presented with the two foils actuated in-phase (**top**) and in anti-phase (**bottom**). The camera is placed at a fixed position below the water tank, and the swimming direction is directed toward the top of the figure. Time in these frame sequences goes from left to right, with an interval between frames of 0.24 s. The initial acceleration phase is not shown. The time stamp on each frame is counted with respect to t=0 when the oscillations start from rest. The case shown here corresponds to d/L=0.6 and f=3 Hz. The size of the visualization window is approximately L×2L (0.15 × 0.3 m).

The amplitude of the oscillation was fixed for the present experiments at $\theta_{0}=60^{\circ }$, while the frequency was set at f=2 Hz (except for the visualization of Figure 2, where the frequency was f=3 Hz). The main parameter examined was the synchronization state, determined by the value of ϕ in the actuation signal: ϕ=0 corresponds to the two neighboring swimmers undulating in-phase, whereas ϕ=π corresponds to the anti-phase case. We also varied the side-by-side distance *d* between the two neighboring swimmers over a small range d/L∈[0.4,0.6]. With the aforementioned actuation, the Reynolds number Re=UL/ν based on the foil length *L* and the self-propelled terminal swimming speed *U* (ν being the kinematic viscosity of the fluid) was Re≈7500, and the Strouhal number based on the tip-to-tip amplitude at the tail $St_{A}=fA/U\approx 1$.

### Particle Image Velocimetry

PIV measurements were performed using a LaVision system (DaVis 7.2) with a Quantronix Darwin-Duo Nd:YLF double-cavity pulsed laser (20 mJ, 527 nm) and a Phantom V9 camera at full resolution: 1632 × 1200. Image acquisition was performed at 100 Hz during 10 s, which was enough time for the self-propelled swimmers to accelerate and leave the measurement window (265 × 154 mm). The laser sheet (of thickness ≈1 mm) was placed at mid-span of the foils and illuminating the foil from the trailing edge. The PIV computation was performed using a multi-pass algorithm with final interrogation windows of 32 × 32 pixels${}^{2}$ and 50% overlap. A standard median filter to remove bad vectors and replace them with second or third correlation peaks was used, and holes in the vector field were filled by interpolation before computing the vorticity fields. Data analysis, post-processing, and visualization were performed using MATLAB and the PIVMat toolbox.

## 3. Results

### 3.1. Cruising Speed

**Figure 3:**
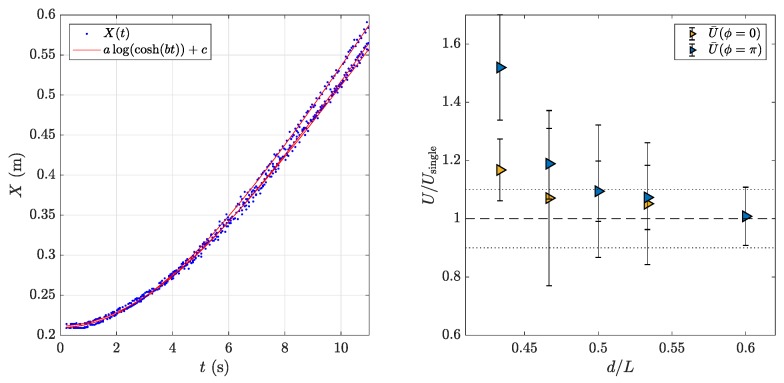
(**left**) Tracking of a typical swimming trajectory. Three different runs are shown with the fit used to compute the final velocity, i.e., the slope of the trajectory at the end of the run. (**right**) Final average cruising velocity of the two foils normalized by the velocity of a foil swimming alone plotted as a function of the separation between the foils and of the synchronization phase.

Figure 2 presents a sequence of snapshots for the two synchronization states. The camera was fixed in the laboratory frame (bottom view from the water tank), so that the self-propelled motion can be seen from the displacement of the pair of foils. Each run started from rest with both foils parallel. In the sequences in Figure 2, the initial acceleration is not shown, but the time stamp on each frame is counted with respect to t=0 when the oscillations started. To examine swimming performance, we used the terminal cruising velocity of the foils. Each run consisted of the pair of foils starting from rest on an aligned side-by-side configuration when the actuation started. Each foil accelerated independently and moved along the *x* swimming direction enforced by the air-bearing rail that supported it, accelerating until the thrust produced by the undulating motion was on average balanced by drag and a terminal cruising velocity was reached. Figure 3 (left) shows three realizations of a typical swimming trajectory for a single foil. We used the analytical function:

$$X_{\mathrm{model}}\left(t\right)=a\log\left(\cosh\left(bt\right)\right)+c\;,$$

obtained considering a self-propelled swimmer driven by a thrust force *F* and subjected to quadratic drag (see [23,26]) to fit the experimental points. As shown in Figure 3 (left), the analytical model described the observed trajectory remarkably well. The terminal velocity for each swimmer can be thus obtained as:

$$U_{\mathrm{single}}=\frac{dX}{dt}{|}_{t\to \infty }\approx ab\;.$$

Since both swimmers were independent, we used the average of the velocities computed as described above for each of the two swimmers to define the cruising velocity of the pair $U=\frac{1}{2}\left(U_{\mathrm{left}}+U_{\mathrm{right}}\right)$. Figure 3 (right) shows a plot of $U/U_{\mathrm{single}}$ for different values of the lateral spacing between the swimmers *d* normalized by the foils’ length *L* and the two prescribed synchronizations. Each point in Figure 3 (right) is the average of three trials, the error bars representing the standard deviation. The large error bars in the measured values of $U/U_{\mathrm{single}}$ were due to the two swimmers being independent, so that even slight experimental perturbations determined that there was always one of the two swimmers that swam slightly faster than the other. The main observation was nonetheless clear: swimming with a neighbor was beneficial in terms of swimming performance for the pair of swimmers, with a clear advantage for swimming with an anti-phase synchronization (ϕ=π).

### 3.2. Flow Field Measurements

**Figure 4:**
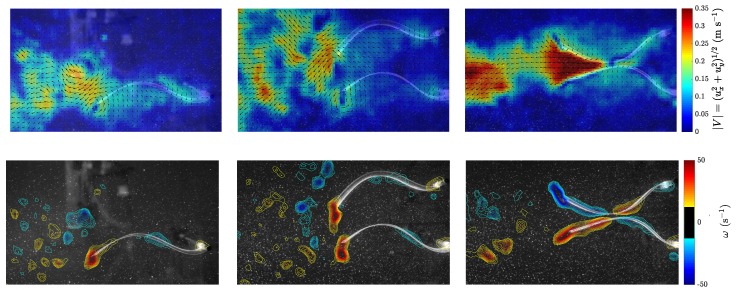
(**Top** row) Snapshot of a typical instantaneous velocity field for a single swimmer (left) and a pair of swimmers in phase (ϕ=0) and anti-phase (ϕ=π) (middle and right frames, respectively). (**Bottom** row) Corresponding vorticity fields. The field of view of the PIV windows is 265 × 154 mm.

We examine now the flow field in the horizontal (xy) symmetry plane of the swimmers at mid-span (z=W/2), using the output of the PIV measurements $u\left(x,y\right)=\left(u_{x},u_{y}\right)$. A snapshot of the velocity field for a single swimmer and the equivalent cases for a pair of swimmers in in-phase (ϕ=0) and anti-phase (ϕ=π) synchronizations is presented in Figure 4, together with the corresponding fields of vorticity ω=∇×u. In the single swimmer case, the velocity field showed the backward jet flow associated with thrust production, while the vorticity field showed the cases with two swimmers. It is remarkable that the two different synchronizations produced very different flow fields: the in-phase case can be qualitatively understood as the superposition of two single swimmers, which could be expected; at the time chosen for the snapshot in Figure 4, each foil was shedding a vortex rotating counterclockwise, and the backward jet was just more spread laterally than the case of the single swimmer. On the contrary, in the anti-phase case, the interaction between the flow produced by the two foils intensified dramatically the backward jet (see the velocity field), and the vortex shedding was symmetrized by the simultaneous shedding of a couple of counter-rotating vortices (see the vorticity field).

**Figure 5:**
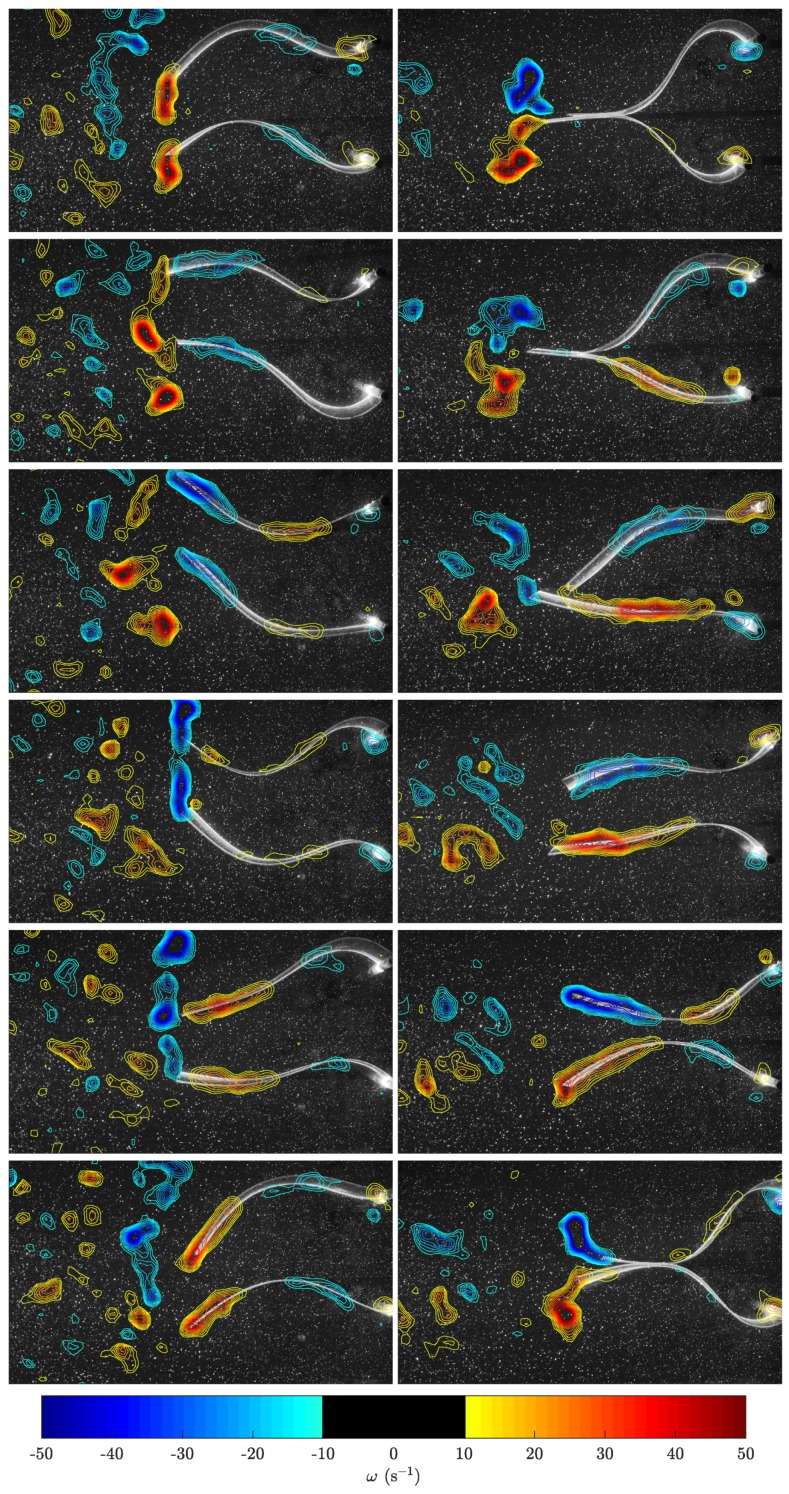
Sequence of vorticity fields over one period of undulation for in-phase (ϕ=0, **left** column) and anti-phase (ϕ=π, **right** column) synchronizations. The field of view of the PIV windows is 265 × 154 mm.

In order to see the time evolution of the flow structures, Figure 5 presents a sequence of snapshots of the vorticity field over one period of undulation for in-phase (ϕ=0) and anti-phase (ϕ=π) synchronizations (the corresponding videos are available as Supplementary Material). As mentioned above, the main vortices shed at the trailing edge with each oscillation were co-rotating in the in-phase case and counter-rotating in the anti-phase case. The main effect in the in-phase case was an undulating flow field that widened the lateral extension of the wake, whereas in the anti-phase case, the two counter-rotating vortices produced by the two neighboring swimmers gave rise to a coherent pulsating jet flow between them.

**Figure 6:**
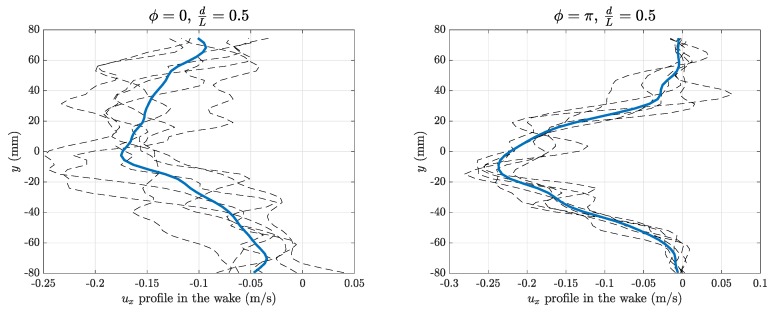
Velocity profiles $u_{x}\left(y\right)$ in the wake of the swimmers over one period (dashed lines) and their average ${\bar{u}}_{x}\left(y\right)$ (solid line) for in-phase (ϕ=0, left) and anti-phase (ϕ=π, right) synchronizations.

**Figure 7:**
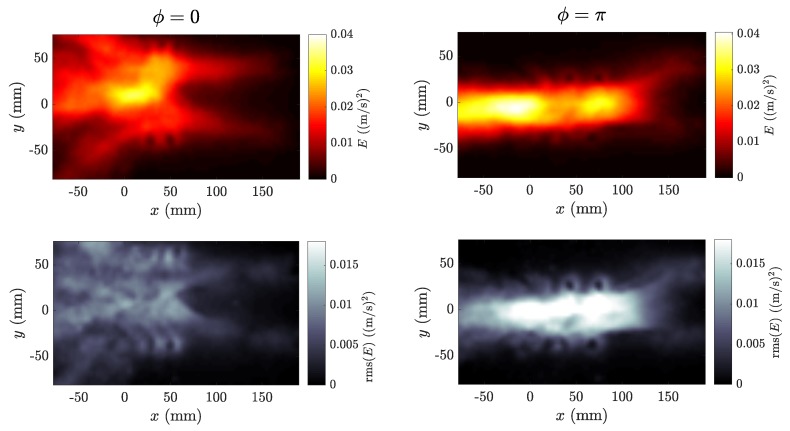
Time average (**top**) and rms (**bottom**) of the kinetic energy field $E_{kin}=\frac{1}{2}\left(u_{x}^{2}+u_{y}^{2}\right)$ over the full run for in-phase (ϕ=0, **left**) and anti-phase (ϕ=π, **right**) synchronizations.

A global quantitative picture of the previous flow features is presented in Figure 6 and Figure 7, which show, respectively, a sequence of $u_{x}\left(y\right)$ profiles measured just behind the swimmer together with their average profile ${\bar{u}}_{x}\left(y\right)$ over one period and a time average of the kinetic energy $E_{kin}=\frac{1}{2}\left(u_{x}^{2}+u_{y}^{2}\right)$ for the full run, for the in-phase and anti-phase cases of Figure 5.

## 4. Discussion

The results shown in Figure 3, where a clear advantage appears for the anti-phase synchronization (ϕ=π), can be readily understood invoking an interpretation in terms of the spatial arrangement of the vortices shed by the foils. Indeed, anti-phase synchronization has been linked to a locomotory advantage in pairs of swimmers using 2D models based on vortex wakes [27,28] and experimental observations of simple models with side-by-side flapping propellers [26,29]. As mentioned above, when flapping in anti-phase, the vortices shed by the two neighboring swimmers each half-period are counter-rotating. They couple as a dipole when the two foils flap toward each other, producing a pulsating jet flow (see the instantaneous velocity field in Figure 4). On the 2D symmetry plane where the PIV measurements were performed, this symmetric vortex shedding recalled the propulsive mechanisms found in nature in axisymmetric animals such as squid or jellyfish: a coherent jet whose alignment determines the swimming velocity. In the in-phase case, where co-rotating vortices are produced during the flapping of the two neighbors, the main difference is that the average propulsive jet is much more spread laterally, and much of the momentum flux is thus directed laterally, so not contributing to the propulsion (see also Figure 6). Although the momentum flux induced by each independent swimmer was on average the same, the phase difference ruled the interference between the propulsive vortex streets produced by each swimmer, determining that flapping in anti-phase was more effective for the pair. A global view of the different effects obtained by in-phase and anti-phase synchronization can be seen by examining the kinetic energy in the area traversed by the swimmers (see Figure 7). The “hot” zones in this average picture are clearly different in the two cases: a spread wake in the in-phase case vs. a coherent energetic zone in the anti-phase case. In spite of the figures showing an average field, the unsteadiness of the flow field can be seen in the outer parts of the wake, where the signature of each foil oscillation and its associated vortex shedding is visible, especially in the rms fields.

The mechanisms described above have been invoked as a possible explanation for the slight preference for anti-phase synchronization observed in pairs of tetra fish as their swimming velocity increases [17], but without any velocimetry measurement in the flow field between the fish. In addition to synchronization, the other crucial parameter was the position of the two neighboring swimmers with respect to each other, which would determine the spatial pattern of the fish school in larger groups. The aforementioned experiments were performed on a shallow swimming channel such that the group of fish adopted a quasi-two-dimensional spatial arrangement. With the same setup, Ashraf et al. [18] showed that the synchronized swimming, in-phase and anti-phase, observed when fish are swimming fast is accompanied by a spatial arrangement where nearest neighbors adopt a phalanx pattern and are thus closer to each other, with a peak in the nearest-neighbor distance (NND) lower than 0.5 body lengths. The effect of the spatial configuration on swimming performance has also been investigated experimentally in a model system considering the propulsive performance of a pair of pitching foils in staggered configurations [29].

The preference of fish for such patterns where nearest neighbors are close to each other and their kinematics are synchronized supports a two-dimensional physical interpretation: in the in-phase case based on the channeling effect [16,27] and in the anti-phase case based on the pulsating jet mechanism described here with the two foil experiment. Indeed, 2D models have been successful in describing other schooling configurations; see, e.g., [30]. However, the thrust production, energy expenditure, and stability of a fish school are very sensitive to the details of the pattern, as shown recently by Li et al. [24] using 3D numerical simulations. Although 2D models and interpretations such as the one we have used to explain the advantage of the anti-phase synchronization can be useful to understand the basic physical mechanisms at play during schooling, the 3D reality needs to be always kept in mind, especially in a bio-inspired robotics perspective.

## Abbreviations

The following abbreviations are used in this manuscript:

PIV	particle image velocimetry
NND	nearest-neighbor distance
2D	two-dimensional
3D	three-dimensional

## References

[1] Vicsek T., Zafeiris A. (2012). Collective motion. Phys. Rep..

[2] Kube C.R., Zhang H. (1993). Collective robotics: From social insects to robots. Adapt. Behav..

[3] Popat R., Cornforth D.M., McNally L., Brown S.P. (2015). Collective sensing and collective responses in quorum-sensing bacteria. J. R. Soc. Interface.

[4] Ramdya P., Lichocki P., Cruchet S., Frisch L., Tse W., Floreano D., Benton R. (2015). Mechanosensory interactions drive collective behavior in Drosophila. Nature.

[5] Halloy J., Sempo G., Caprari G., Rivault C., Asadpour M., Tâche F., Saïd I., Durier V., Canonge S., Amé J.M. (2007). Social integration of robots into groups of cockroaches to control self-organized choices. Science.

[6] Raj A., Thakur A. (2016). Fish-inspired robots: Design, sensing, actuation, and autonomy—A review of research. Bioinspir. Biomim..

[7] Lopez U., Gautrais J., Couzin I.D., Theraulaz G. (2012). From behavioral analyses to models of collective motion in fish schools. Interface Focus.

[8] Filella A., Nadal F., Sire C., Kanso E., Eloy C. (2018). Model of collective fish behavior with hydrodynamic interactions. Phys. Rev. Lett..

[9] Lighthill M.J. (1960). Note on the swimming of slender fish. J. Fluid Mech..

[10] Blevins E., Lauder G.V. (2013). Swimming near the substrate: A simple robotic model of stingray locomotion. Bioinspir. Biomim..

[11] Quinn D.B., Moored K.W., Dewey P.A., Smits A.J. (2014). Unsteady propulsion near a solid boundary. J. Fluid Mech..

[12] Fernández-Prats R., Raspa V., Thiria B., Huera-Huarte F., Godoy-Diana R. (2015). Large-amplitude undulatory swimming near a wall. Bioinspir. Biomim..

[13] Kurt M., Cochran-Carney J., Zhong Q., Mivehchi A., Quinn D.B., Moored K.W. (2019). Swimming freely near the ground leads to flow-mediated equilibrium altitudes. J. Fluid Mech..

[14] Weihs D. (1973). Hydromechanics of fish schooling. Nature.

[15] Hemelrijk C.K., Reid D., Hildenbrandt H., Padding J.T. (2015). The increased efficiency of fish swimming in a school. Fish Fish..

[16] Daghooghi M., Borazjani I. (2015). The hydrodynamic advantages of synchronized swimming in a rectangular pattern. Bioinspir. Biomim..

[17] Ashraf I., Godoy-Diana R., Halloy J., Collignon B., Thiria B. (2016). Synchronization and collective swimming patterns in fish (*Hemigrammus bleheri*). J. R. Soc. Interface.

[18] Ashraf I., Bradshaw H., Ha T.T., Halloy J., Godoy-Diana R., Thiria B. (2017). Simple phalanx pattern leads to energy saving in cohesive fish schooling. Proc. Natl. Acad. Sci. USA.

[19] Collignon B., Séguret A., Chemtob Y., Cazenille L., Halloy J. (2019). Collective departures and leadership in zebrafish. PLoS ONE.

[20] Alben S., Witt C., Baker T., Anderson E., Lauder G. (2012). Dynamics of freely swimming flexible foils. Phys. Fluids.

[21] Dewey P.A., Boschitsch B.M., Moored K.W., Stone H.A., Smits A.J. (2013). Scaling laws for the thrust production of flexible pitching panels. J. Fluid Mech..

[22] Piñeirua M., Thiria B., Godoy-Diana R. (2017). Modelling of an actuated elastic swimmer. J. Fluid Mech..

[23] Raspa V., Ramananarivo S., Thiria B., Godoy-Diana R. (2014). Vortex-induced drag and the role of aspect ratio in undulatory swimmers. Phys. Fluids.

[24] Li G., Kolomenskiy D., Liu H., Thiria B., Godoy-Diana R. (2019). On the energetics and stability of a minimal fish school. PLoS ONE.

[25] Li G., Kolomenskiy D., Liu H., Thiria B., Godoy-Diana R. (2019). On the interference of vorticity and pressure fields of a minimal fish school. J. Aero Aqua-Bio-Mech..

[26] Raspa V., Godoy-Diana R., Thiria B. (2013). Topology-induced effect in biomimetic propulsive wakes. J. Fluid Mech..

[27] Weihs D. (1975). Some hydrodynamical aspects of fish schooling. Swimming and Flying in Nature.

[28] Kanso E., Newton P.K. (2009). Locomotory Advantages to Flapping Out of Phase. Exp. Mech..

[29] Huera-Huarte F.J. (2018). Propulsive performance of a pair of pitching foils in staggered configurations. J. Fluids Struct..

[30] Ramananarivo S., Fang F., Oza A., Zhang J., Ristroph L. (2016). Flow interactions lead to orderly formations of flapping wings in forward flight. Phys. Rev. Fluids.

